# Supplementary material for: WordCommentsAnalyzer: A windows software tool for qualitative research
Source: F1000Res. 2018 Sep 4;7:536. Originally published 2018 May 3. [Version 2] doi: 10.12688/f1000research.14819.2 (PMC6137411; doi:10.12688/f1000research.14819.2)
Supplement: Supplementary file 3 [file f1000research-7-17614-s0002.tgz › b2a79496-4ada-47c4-9682-a99ce867836d.zip!/Eight Iranian Health Journals/Archives of Rehabilitation_6.docx]

© Archives of Rehabilitation (RJ). University of Social Welfare and Rehabilitation Sciences, Koodakyar Alley, Daneshjoo Blv., Evin, Tehran, Iran. This work is licensed under [CC BY-NC 4.0](https://creativecommons.org/licenses/by-nc/4.0/).

Volume & Issue: دوره 17 - شماره 4

Title: بررسی اثرات سن و سرعت راه‌رفتن بر نیروی عکس‌العمل عمودی زمین در جوانان و سالمندان

http://rehabilitationj.uswr.ac.ir/article-1-1878-fa.html

هدف افزایش پیش‌رونده جمعیت سالمندان در بسیاری از کشورهای توسعه‌یافته توجه را بیش‌ازپیش به سلامت این گروه سنی معطوف کرده است. یکی از مهم‌ترین فعالیت‌های حرکتی زندگی روزانه راه‌رفتن است که نقشی حیاتی در زندگی افراد این گروه سنی ایفا می‌کند و می‌تواند به تأمین سلامت جسمانی و روانی سالمندان کمک شایانی کند. به‌طورخاص بررسی نیروی عکس‌العمل زمین در حین راه‌رفتن سالمندان در سرعت‌های مختلف و مقایسه آن با جوانان می‌تواند درک بهتری از سازوکارهای زیربنایی تنظیم سرعت در طول راه‌رفتن به‌وجود آورد. بنابراین هدف پژوهش حاضر بررسی اثرات سن و سرعت راه‌رفتن بر نیروی عکس‌العمل عمودی زمین در جوانان و سالمندان بود. روش بررسی آزمودنی‌های این پژوهش را پانزده مرد جوان و پانزده مرد سالمند تشکیل می‌داد که میانگین سنی آن‌ها به‌ترتیب 46/26 و 3/70 سال بود. همگی این افراد سالم و به روش نمونه‌گیری غیرتصادفی انتخاب شده بودند. یک مسیر پیاده‌روی ده‌متری در طول آزمایشگاه درنظرگرفته و یک صفحه نیروی سه‌محوره کیستلر در وسط مسیر جاسازی شد. فاصله نقطه شروع راه‌رفتن آزمودنی‌ها تا صفحه نیرو چهار متر بود. هریک از آزمودنی‌ها این مسیر ده‌متری را با سرعت‌های مختلف خودانتخابی (100درصد) و کُند (80درصد) و سریع (120درصد) راه رفتند. نیروهای عکس‌العمل عمودی زمین طی مراحل استقرار راه‌رفتن شامل مرحله پذیرش وزن یا اوج اول (FZ1) و مرحله میانه استقرار یا عمق (FZ2) و مرحله پیشروی یا اوج دوم (FZ3) با صفحه نیرو با فرکانس 200 هرتز ثبت شد. داده‌‌ها با آزمون تحلیل واریانس با اندازه‌گیری‌های مکرر و آزمون تعقیبی بونفرونی تجزیه‌وتحلیل شد. یافته‌ها نتایج نشان داد اثر سن (002/0=P) و سرعت (001/0=P) بر میانگین نیروی عکس‌العمل عمودی زمین معنی‌دار است. همچنین اثر تعاملی سن و سرعت بر میانگین نیروی عکس‌العمل عمودی زمین نیز معنی‌دار بود (013/0=P). مطابق آزمون بونفرونی در هر دو گروه سنی جوانان و سالمندان میانگین نیروی عکس‌العمل عمودی بین سرعت‌های مختلف تفاوت معنی‌داری داشت (01/0>P). به‌علاوه در سرعت‌های 100درصد (036/0=P) و 120درصد (002/0=P) بین جوانان و سالمندان تفاوت‌‌های معنی‌‌داری دیده شد. در سرعت 80درصد در نیروهای اوج اول و عمق و اوج دوم عکس‌العمل عمودی زمین بین جوانان و سالمندان تفاوت معنی‌داری وجود نداشت (05/0P). این در حالی است که در نیروی عمق تفاوت معنی‌داری وجود نداشت (05/0P). نتیجه‌گیری به‌طورخلاصه نتایج پژوهش حاضر نشان داد هنگام راه‌رفتن با سرعت‌های دلخواه و سریع نیروهای اوج اول و دوم در بین جوانان و سالمندان تفاوت معنی‌داری دیده شد؛ درحالی‌که در نیروی عمق تفاوت معنی‌داری وجود نداشت. علاوه‌براین در هر دو گروه سنی در مقدار نیروهای اوج اول و دوم بین سرعت‌های مختلف راه‌رفتن تفاوت‌های معنی‌داری مشاهده شد. از نتایج تحقیق حاضر چنین استنباط می‌شود که کاهش نیروی اوج اول عکس‌‌العمل عمودی زمین در سالمندان به‌نوعی مؤید ضعف پویا و واکنش سیستم عصبی‌عضلانی در آن‌ها و کاهش نیروی اوج دوم عکس‌العمل زمین بیانگر عملکرد ضعیف‌تر عضلات پلانتارفلکسور سالمندان در هنگام راه‌رفتن است. به‌نظر می‌‌رسد تقویت عضلات اندام تحتانی در سالمندان بتواند به بهبود سرعت راه‌رفتن آن‌ها کمک کند و در کاهش خطرهای افتادن و ناتوانی عملکردی مؤثر واقع شود.

Title: مقایسه کیفیت زندگی بزرگ‌سالان دچار لکنت با افراد بدون‌لکنت با تأکید بر شدت لکنت

http://rehabilitationj.uswr.ac.ir/article-1-1869-fa.html

هدف نتایج مطالعات و تجارب افراد مبتلا به لکنت نشان داده است که لکنت می‌تواند مشکلات روانی‌اجتماعی را به‌وجود آورد که به‌طورمنفی بر کیفیت زندگی تأثیر می‌گذارد. آزمون OASES-A یک آزمون جامع روان‌شناختی مبتنی‌بر چهار شاخصه عمده است و در شاخصه چهارم این آزمون روی دلالت بر کیفیت زندگی افراد تأکید شده است. هدف پژوهش حاضر بررسی مقایسه کیفیت زندگی در افراد دچار لکنت با افراد بدون‌لکنت و بررسی همبستگی شدت لکنت با هریک از مؤلفه‌های آزمون OASES-A در افراد مبتلا به لکنت بود. روش بررسی مطالعه مقطعی توصیفی‌تحلیلی حاضر روی 56 فرد بزرگ‌سال دچار لکنت و 56 فرد بزرگ‌سال فاقد لکنت انجام شد که براساس سن، جنس، میزان تحصیلات و شغل با گروه هدف همتاسازی شده بودند. در این مطالعه نمونه‌گیری به‌صورت نمونه‌گیری طبقه‌ای‌تصادفی انجام گرفت. برای جمع‌آوری داده‌ها آزمون تعیین شدت لکنت SSI-3 در موقعیت گفتاری خواندن شفاهی متن دویست‌کلمه‌ای (448هجایی) و برای بررسی کیفیت زندگی از آزمون ارزیابی جامع تجربه فرد از لکنت بزرگ‌سال به‌کاررفت. افزون‌براین از آزمون تی مستقل برای مقایسه میانگین‌های کیفیت زندگی و سن و از آزمون کای اسکوئر به‌منظور مقایسه میانگین‌های جنسیت و سطح تحصیلات و شغل دو گروه دچار لکنت و بدون‌لکنت و از آزمون‌های لون و ضریب همبستگی اسپیرمن برای بررسی ارتباط کیفیت زندگی با شدت لکنت استفاده شد. داده‌ها به کمک نسخه شانزدهم نرم‌افزار SPSS تجزیه‌وتحلیل شد. یافته‌ها نتایج نشان داد که میانگین نمرات کیفیت زندگی افراد دچار لکنت در مقایسه با افراد بدون‌لکنت بیشتر و این تفاوت از نظر آماری معنادار بود (001/0>P). همچنین همبستگی معنی‌داری بین شدت لکنت با کیفیت زندگی یافت شد (001/0>P). علاوه‌براین از لحاظ سن (68/0 نتیجه‌گیری براساس شاخصه‌های آزمون ارزیابی جامع تجربه فرد از لکنت افراد دچار لکنت سطح کیفیت زندگی پایین‌تری داشتند. بررسی توزیع امتیازهای کیفیت زندگی بیماران دچار لکنت در شدت‌های مختلف آن نشان داد کیفیت زندگی افراد مبتلا به لکنت در مراحل خفیف و متوسط بهتر از مراحل شدید و خیلی شدید بود. به‌علاوه نتایج تحقیق نشان داد متغیرهای سن، جنس، میزان تحصیلات و شغل عوامل مؤثری بر کیفیت زندگی بود. بنابراین در فرایند ارزیابی و درمان لکنت بزرگ‌سال شایسته است ضمن توجه به جنبه‌های روانی گفتار، در زمینه‌های روان‌شناختی تغییرات مثبت ایجاد شود و برای برقراری تعاملات ارتباطی روزمره بهتر و ارتقای کیفیت زندگی افراد نگرش آنان نیز مدنظر قرار گیرد.

Title: بررسی تأثیر آموزش مهارت‌های اجتماعی بر مشارکت اجتماعی معلولان جسمی‌وحرکتی مجتمع آموزشی نیکوکاری رعد مرکز

http://rehabilitationj.uswr.ac.ir/article-1-1946-fa.html

هدف معلولیت مفهومی کلی است که با محرومیت‌هایی که فرد در نتیجه نقص (اختلال) و ناتوانی تجربه می‌کند مرتبط است. هر گونه کمبود و کاهش فرصت برای مشارکت در زندگی اجتماعی در سطحی برابر با دیگران معلولیت نامیده می‌شود. با توجه به افزایش جمعیت معلولان جسمی و حرکتی در اثر عوامل ارثی و مادرزادی و اکتسابی (ضربه، تصادف، بیماری و سالمندی) و محدودیت ایجادشده در اثر معلولیت و بحث مشارکت اجتماعی معلولان در طبقه بندی ICF، پرداختن به مشارکت اجتماعی معلولان جسمی و حرکتی اهمیت خود را نشان می‌دهد. با توجه به مطالعات صورت‌گرفته توسط پژوهشگران، ازجمله نیازهای معلولان، نیاز آموزشی و کسب مهارت‌های لازم برای حضور فعال در جامعه است. لذا کمک به معلولان جسمی و حرکتی به منظور رشد و گسترش مهارت‌های زندگی مطلوب، ایجاد یا افزایش اعتماد به نفس در برخورد با مشکلات، کمک به آن‌ها در رشد و تکامل عواطف و مهارت‌های اجتماعی برای مشارکت اجتماعی موفق ضروری به نظر می‌رسد. لذا هدف از پژوهش حاضر، بررسی اثربخشی آموزش مهارت‌های اجتماعی بر مشارکت اجتماعی معلولان جسمی و حرکتی مجتمع آموزشی نیکوکاری رعد مرکز بود. روش بررسی پژوهش حاضر از نوع شبه‌آزمایشی با طرح پیش‌آزمون و پس‌آزمون با گروه کنترل بود. جامعه آماری پژوهش تمام کارآموزان مرکز آموزشی نیکوکاری رعد مرکز بودند که در زمان انجام پژوهش کل کارآموزان مرکز ۱۹۵ نفر بود. از این بین ۱۲۰ نفر زن و ۷۵ نفر مرد بودند. نمونه بررسی‌شده در این تحقیق 60 نفر از معلولان جسمی و حرکتی مرکز آموزشی نیکوکاری رعد مرکز شهر تهران بودند که با توجه به معیارهای ورود به روش تصادفی ساده انتخاب و با قرعه‌کشی به دو گروه مداخله و کنترل تقسیم شدند. برنامه آموزش مهارت‌های اجتماعی در هشت جلسه و هر جلسه 90 دقیقه برای گروه آزمایش انجام گرفت. به منظور سنجش مشارکت اجتماعی افراد قبل از شروع اولین جلسه و بعد از پایان آخرین جلسه بین افراد شرکت‌کننده در پژوهش پرسش‌نامه مشارکت اجتماعی و پرسش‌نامه جمعیت‌شناختی توزیع و توسط آنان تکمیل شد. این پرسش‌نامه را یزدان پناه در سال ۱۳۸۶ در ایران ساخته و روا و پایا شده است. ضرایب روایی و پایایی به‌دست‌آمده برای مشارکت اجتماعی 788/0 و ۸۷/۰ بود. برای تحلیل داده‌ها از نسخه 16 نرم‌افزار SPSS، آمار توصیفی نظیر میانگین و فراوانی و انحراف‌معیار و آمار استنباطی نظیر آزمون‌های من‌ویتنی، لون، کولموگروف اسمیرنف، تی وابسته و مستقل و آزمون کوواریانس آنوا استفاده شد. یافته‌ها نتایج این پژوهش نشان داد بین مشارکت اجتماعی کل و خرده‌مقیاس‌های مشارکت نهادی و غیرنهادی افراد قبل و بعد از آموزش مهارت‌های اجتماعی (p<0/05) و نیز بین مشارکت اجتماعی کل و زیرمقیاس‌های آن در بین افراد آموزش‌دیده و آموزش‌ندیده (p<0/05) تفاوت آماری معناداری وجود دارد. نتیجه‌گیری با توجه به نتایج این مطالعه می‌توان گفت برگزاری جلسه‌های آموزش مهارت‌های اجتماعی بر مشارکت اجتماعی معلولان جسمی و حرکتی در دو بُعد نهادی و غیرنهادی مؤثر است. لذا احتمال داده می‌شود که به‌کارگیری گسترده‌تر این نوع مداخله توسط متخصصان حوزه معلولان بتواند در بر طرف‌کردن محدودیت مشارکت معلولان جسمی و حرکتی مؤثر واقع شود.

Title: بررسی تأثیر کفی سفارشی ساخته‌شده با شیوه جدید بر میزان درد و عملکرد در زنان مبتلا به صافی کف پا انعطاف‌پذیر: مطالعه مقدماتی

http://rehabilitationj.uswr.ac.ir/article-1-1922-fa.html

هدف صافی کف پا به کاهش یا ازبین‌رفتن قوس طولی داخلی کف پا گفته می‌شود و در این حالت پا در وضعیت پرونیشن قرار می‌گیرد. عارضه صافی کف پا اغلب اختلال پیچیده‌ای است که علائم متنوع و درجات مختلفی از بدشکلی و ناتوانی دارد. گاهی این اختلال می‌تواند به‌علت تغییر در الایمنت مشکلاتی در مفصل مچ پا و زانو به‌وجود آورد و روی مفاصل اندام تحتانی و کمر استرس وارد و ایجاد درد کند. همچنین این عارضه می‌تواند سبب اعمال فشار بر بافت‌های عمقی‌تر مانند لیگامان‌ها و تاندون‌ها نیز شود. عارضه صافی کف پا به دو نوع انعطاف‌پذیر و سخت تقسیم می‌شود. شیوع این عارضه به عوامل مختلفی بستگی دارد. تحقیقات نشان می‌دهد صافی کف پا احتمال آسیب‌دیدن پا را حین فعالیت‌های روزانه افزایش می‌دهد. درمان‌های محافظتی به‌طور شایع برای درمان افراد مبتلا به صافی کف پا استفاده می‌شود. ازجمله این درمان‌ها می‌توان به درمان‌های ارتوزی و فیزیوتراپی اشاره کرد. متخصصان کفی‌ها را عموماً برای درمان صافی کف پا تجویز می‌کنند. معمولاً کفی‌ها قوس کف پا را به حالت معمولی برمی‌گرداند و عوارض ناشی از صافی کف پا را کاهش می‌دهد. کفی با شیوه medial heel skive یکی از روش‌های درمانی است.  روش بررسی این مطالعه از نوع شبه‌تجربی به‌صورت قبل و بعد بود که در آن 12 زن مبتلا به صافی کف پا از میان دانشجویان دانشگاه علوم بهزیستی و توانبخشی شرکت داشتند. معیارهای ورود و خروج برای این افراد درنظر گرفته شد. برای به‌دست‌آوردن قالب منفی، پای هر فرد قالب‌گیری شد. برای نخستین‌بار روش اصلاح medial heel skive را کربی معرفی کرده است و مطابق با آن اصلاحات قالب مثبت انجام شد. سپس به هر فرد کفی ساخته‌شده با شیوه medial heel skive داده شد. معیارهای درد و عملکرد به‌ترتیب با ابزار‌های مقیاس آنالوگ بینایی (VAS) و FAOS قبل از استفاده از کفی و پس از 6 هفته استفاده از کفی مدنظر سنجیده شد. مقیاس آنالوگ بینایی شامل یک خط افقی ۱۰۰ میلی‌متری است که میزان درد را با آن اندازه می‌گیرند. پرسش‌نامه مدنظر نیز شامل ۲۱ سؤال است که میزان عملکرد را ارزیابی می‌کند. برای مقایسه میزان درد و عملکرد افراد مبتلا به صافی کف پا قبل و بعد از استفاده از کفی با شیوه medial heel skive، روش آماری تی زوجی و آزمون ناپارامتری به‌کاررفت.  یافته‌ها در این مطالعه نشان داده شد که میزان درد و عملکرد در افراد مبتلا به صافی کف پا بعد از 6 هفته استفاده از کفی ساخته‌شده به‌شیوه medial heel skive به‌طور معناداری بهبود یافت (P<0/001).  نتیجه‌گیری با توجه به نتایج مطالعه، در عملکرد و درد افراد مبتلا به صافی کف پا با استفاده از کفی با شیوه medial heel skive تفاوت معناداری وجود داشت؛ بنابراین به‌نظر می‌رسد این کفی تجویز مناسبی برای بهبود علایم در افراد مبتلا به صافی کف پاست.

Title: اثربخشی برنامه جامع توانبخشی والدمحور نوایش بر رشد مهارت‌های ارتباطی اولیه و زبانی کودکان ناشنوای 0 تا 2 سال

http://rehabilitationj.uswr.ac.ir/article-1-1876-fa.html

هدف مداخله زودهنگام اقدامی ضروری برای پیشگیری از تأثیر ناشنوایی بر رشد زبان و مهارت‌های ارتباطی است. تشخیص زودهنگام ناشنوایی و برنامه‌های منسجم توانبخشی نقش بسزایی در رشد زبان بیانی و زبان دریافتی و مهارت‌های ارتباطی کودکان ناشنوا ایفا می‌کند. از طرفی کاهش سن تشخیص و مداخله ضرورت حضور بیشتر والدین در برنامه‌های توانبخشی را به‌دنبال دارد. افزون‌براین ارتباط کودکان ناشنوا با والدین در سال‌های اولیه در رشد زبان و مهارت‌های ارتباطی مؤثر است. اهمیت مشارکت والدین در توانبخشی کودکان ناشنوا به‌عنوان والد-درمانگر، لزوم ارائه برنامه جامع توانبخشی خانواده‌محور را هم‌زمان با تشخیص ناشنوایی و شروع اقدامات ادیولوژیکی و اتولوژیکی نشان می‌دهد. مطالعه حاضر با هدف بررسی اثربخشی برنامه جامع توانبخشی والدمحور نوایش بر رشد مهارت‌های ارتباطی اولیه و زبانی کودکان ناشنوای 0 تا 2 سال انجام شده است. روش بررسی پژوهش حاضر از نوع نیمه‌آزمایشی بود و در آن از طرح پیش‌آزمون-پس‌آزمون با گروه گواه استفاده شد. با روش نمونه‌گیری هدفمند، 62 نفر از کودکان ناشنوای 0 تا 2 سال (30 دختر و 32 پسر) از مرکز کاشت حلزون شنوایی بیمارستان رسول‌اکرم(ص) به‌صورت هدفمند انتخاب و به‌طورتصادفی در دو گروه آزمایش و گواه قرار گرفتند. روی این کودکان عمل کاشت حلزون شنوایی انجام گرفته بود یا برای استفاده از کاشت حلزون شنوایی نامزد شده و در مرکز کاشت حلزون شنوایی بیمارستان رسول‌اکرم(ص) تشکیل پرونده داده بودند. آزمودنی‌های گروه آزمایش برنامه جامع توانبخشی والدمحور نوایش را در قالب 18 جلسه گروهی و فردی (6 جلسه گروهی و 12 جلسه فردی) دریافت کردند. همچنین در طول اجرای تحقیق، برای پیگیری روند اجرای برنامه و نیز پاسخ‌گویی به سؤالات والدین مشاوره‌های تلفنی انجام گرفت. برای بررسی مهارت‌های ارتباطی اولیه و زبانی از ابزارهایی مانند ویرایش سوم پرسش‌نامه سنین و مراحل (ASQ-3) و نیم‌رخ مقیاس رشدی رفتار نمادین و ارتباط ((CSBSDP استفاده شد. تجزیه‌وتحلیل داده‌ها با نسخه 19 نرم‌افزار SPSS و در سطح معنی‌داری 001/0>P انجام گرفت. همچنین برای تحلیل یافته‌ها از تحلیل کواریانس تک و چندمتغیری استفاده شد. یافته‌ها یافته‌ها نشان داد میانگین نمرات در حیطه مهارت ارتباطی در گروه آزمایش در مقایسه‌با گروه کنترل در پس‌آزمون در مقایسه‌با پیش‌آزمون افزایش بیشتری یافته است (33/43). همچنین تحلیل یافته‌ها با استفاده از تحلیل کواریانس تک‌متغیری و چندمتغیری حاکی از آن بود که برنامه جامع توانبخشی نوایش در افزایش نمرات در حیطه مهارت‌های ارتباطی (23/26)، زبان بیانی و گفتار (83/16) و رفتار نمادین (77/12) مؤثر بوده است (001/0>P). نتیجه‌گیری نتایج این پژوهش حاکی از تأثیر برنامه جامع توانبخشی والدمحور نوایش بر رشد مهارت‌های ارتباطی اولیه و زبان در کودکان ناشنواست. ازاین‌رو کاربرد این برنامه در مراکز توانبخشی کودکان ناشنوا و با هدف آموزش والدین به‌عنوان درمانگر اصلی کودک ناشنوا توصیه می‌شود.
